# Placental Related Disorders of Pregnancy 2.0

**DOI:** 10.3390/ijms241814286

**Published:** 2023-09-19

**Authors:** Eun D. Lee, Hiten D. Mistry

**Affiliations:** 1 Department of Microbiology and Immunology, School of Medicine, Massey Cancer Center, Virginia Commonwealth University, Richmond, VA 23298, USA; eun.lee@vcuhealth.org; 2 Department of Women and Children’s Health, School of Life Course Sciences, King’s College London, London SE5 9NU, UK

Following our first Special Issue, we are pleased to present this Special Issue in the *International Journal of Molecular Sciences* entitled ‘Placental Related Disorders of Pregnancy 2.0′ [1]. The placenta, an extraordinary organ, forms outside the embryo and is connected through a cord of vessels. Its development arises from intricate interactions between fetal and maternal tissues within the pregnant uterus. Unlike stable, mature adult organs, the placenta adapts and performs diverse functions throughout development, making it a dynamically evolving organ. Its primary purpose is to maintain a protected environment for the undisturbed growth and development of the embryo/fetus.

Placental-related disorders are mainly exclusive to humans and affect approximately one-third of pregnancies. These disorders can lead to higher maternal and fetal mortality and morbidity rates, with long-term health consequences for both the mother and child. Changes in human lifestyle, such as delayed childbirth and hypercaloric diets, may have contributed to the increased global incidence of placental-related disorders in recent decades.

This Special Issue is a compilation of 12 research manuscripts and reviews covering all aspects of placentation and its related disorders. The first two systematic reviews investigate pregnancy latency biomarkers after preterm premature rupture of membranes [2] and atypical pre-eclampsia before 20 weeks of gestation [3]. This is followed by detailed reviews of the apelinergic system in pregnancy [4]; serum screen markers for trisomy 21 and the crucial involvement of the placenta [5]; how the placenta can provide a protective role in SARS-CoV-2 transplacental transmission [6]; endoplasmic reticulum aminopeptidase 1 and 2 and their involvement in pregnancy and cancer [7]; and how rodent and rabbit models can be used in pre-eclampsia research [8]. Moreover, this Special Issue also contains original articles with novel data covering hypothyroidism [9] and pre-eclampsia/fetal growth restriction [10,11,12,13].

This second Special Issue focuses on placental research, utilising diverse, well-established, and cutting-edge techniques to present novel and current data. The aim is to deepen and streamline our comprehension of placentation, the mechanisms responsible for adverse outcomes in pregnancy, and the potential long-term risks of complications.
